# Comparative Transcriptomics Reveals Divergent Expression Patterns Related to Carbon Metabolism, Transport, and Regulation in Juvenile Stems of Four Fagaceae Species

**DOI:** 10.3390/genes17091139

**Published:** 2026-09-17

**Authors:** Xiyuan Yang, Liwen Wu, Yangdong Wang

**Affiliations:** 1 State Key Laboratory of Tree Genetics and Breeding, Chinese Academy of Forestry, No. 1 Dongxiaofu, Xiangshan Road, Haidian District, Beijing 100091, China; yangxiyuan_as@163.com (X.Y.); wuliwenhappy@163.com (L.W.); 2 Research Institute of Subtropical Forestry, Chinese Academy of Forestry, No. 73 Daqiao Road, Fuyang District, Hangzhou 311400, China

**Keywords:** Fagaceae, juvenile stem, comparative transcriptomics, photosynthesis, sugar allocation, cell wall remodeling, ABC transport, qRT-PCR

## Abstract

**Background/Objectives:** We compared juvenile whole-stem transcriptomes of *Quercus glauca*, *Quercus acutissima*, *Quercus fabri*, and *Castanopsis sclerophylla* to examine carbon metabolism and transport. **Methods:** Three seedlings per species were sampled in one session. RNA sequencing (RNA-seq) data were analyzed using differential-expression analysis, direction-specific Kyoto Encyclopedia of Genes and Genomes (KEGG) enrichment, gene set enrichment analysis (GSEA), and leading-edge analysis. Quantitative reverse-transcription polymerase chain reaction (qRT-PCR) was performed on aliquots from the same RNA extractions. **Results:** Reference-based analyses identified 8596–11,823 differentially expressed genes across six contrasts. GSEA detected 25 significant pathway-by-contrast associations across 13 pathways at a false discovery rate below 0.05. *Q. glauca* showed photosynthesis, starch turnover, and flavonoid pathway signals. *Q. acutissima* showed sugar-metabolism and ATP-binding cassette (ABC) transport signals, with *XTH23* providing an annotation-based wall-remodeling candidate. *Q. fabri* showed enrichment of sulfur amino acid metabolism, nucleotide sugar metabolism, and mitogen-activated protein kinase signaling. *C. sclerophylla* showed ABC transport and galactose metabolism enrichment, with candidates annotated for raffinose synthesis and extracellular sucrose cleavage. Eleven of 12 candidates were present in at least one significant GSEA leading edge; *XTH23* was selected using differential expression and annotation. Both assays identified the same species as having the highest expression for every candidate; eight genes also showed the same rank order across all four species. **Conclusions:** The sampled stems differed in the expression of genes associated with carbon metabolism, transport, and regulation. These whole-stem patterns provide candidates for tissue-resolved and physiological studies.

## 1. Introduction

Juvenile stems are sites of transport, storage, secondary growth, and structural reinforcement. Their living tissues also respire, exchange metabolites, and, when exposed to light, reassimilate internally released CO_2_ [1,2,3,4,5]. Carbon entering a stem is therefore allocated to maintenance, temporary storage, long-distance transport, cell-wall formation, and defense-related metabolism. The balance among these processes influences how a young tree builds vascular tissue and supports future growth.

Fagaceae species differ in leaf habit, wood development, ecological distribution, and stress responses. Comparing the three *Quercus* species allows differences within a genus to be examined, whereas including *Castanopsis sclerophylla* extends the comparison across genera within Fagaceae [6]. RNA sequencing (RNA-seq) provides a genome-wide view of gene expression [7]. Studies in woody plants have examined xylem differentiation, stem growth, compression wood, organ size, drought responses, and genotype-by-environment variation [8,9,10,11,12,13,14,15,16,17].

Within Fagaceae, transcriptomic studies have linked carbon metabolism to both development and stress responses. Combined transcriptome and proteome analyses linked sucrose synthesis to *Quercus fabri* axillary-bud outgrowth [14]. In a drought and rewatering study, *Quercus acutissima* showed reduced expression of a sucrose synthase-like gene and increased expression of granule-bound starch synthase 1 during drought. *Quercus palustris* showed increased expression of amylase-related genes and reduced expression of granule-bound starch synthase 1, with changes after rewatering [16]. These studies highlight the relevance of sugar synthesis and breakdown to oak growth and stress responses. Here, we examine the expression of genes involved in these processes, alongside transport and wall-related pathways, in juvenile whole stems grown under the same conditions.

Photosynthesis contributes local carbon, whereas starch and sucrose pathways regulate short-term storage and mobilization. Invertases control sucrose cleavage and sink activity, nucleotide sugars provide substrates for wall polysaccharide synthesis, and membrane transporters redistribute sugars, hormones, and specialized metabolites [1,5,18,19,20,21,22,23,24,25,26,27]. Hormone signaling, protein phosphorylation, redox regulation, and transcriptional control further influence carbon allocation and cellular protection [28,29,30,31,32,33,34]. These processes occur across the cortex, phloem, cambium, xylem, pith, and external tissues of the stem.

Here, we compared juvenile whole-stem transcriptomes of four Fagaceae species grown and sampled under the same conditions. We asked which pathways differed among the sampled species, how their expression patterns related to carbon metabolism and transport, and which genes warranted further study. We combined differential-expression analysis, direction-specific enrichment, gene set enrichment analysis (GSEA), and leading-edge analysis. Quantitative reverse-transcription polymerase chain reaction (qRT-PCR) was used to compare expression patterns of 12 candidate genes with those obtained by RNA-seq.

## 2. Materials and Methods

### 2.1. Plant Materials, Experimental Design, and Sampling

Two-year-old, seed-derived seedlings of *Q. fabri* (Qf), *Q. acutissima* (Qa), *Quercus glauca* (Qg), and *C. sclerophylla* (Cs) were maintained in the same greenhouse at the Research Institute of Subtropical Forestry, Chinese Academy of Forestry (Fuyang District, Hangzhou, Zhejiang Province, China). Seeds of all four species originated from Hangzhou. The seedlings were not from named cultivars or selected lines. Species identities were verified on multiple occasions by taxonomic specialists at the institute, and the living source plants remain in the institute collection. All four species were sampled during the same session in July 2025, limiting short-term variation in light, temperature, and time of day.

For each species, three independent seedlings were selected. One stem segment approximately 3 cm long and 0.5 cm in diameter was excised from each seedling at 1 m above ground. The segment included the external protective tissues, cortex, phloem, cambium, xylem, and pith present at that position. Each segment constituted one biological replicate; material was not pooled among seedlings. Sampling at a common height standardized the position along the stem but did not ensure anatomical or developmental equivalence across species. Samples were frozen immediately in liquid nitrogen and kept frozen until RNA extraction. Sample metadata are provided in Table S1.

### 2.2. RNA Extraction and Quality Assessment

The entire frozen stem segment from each seedling was used for one RNA extraction with TRIzol reagent (Invitrogen, catalog no. 15596018, Carlsbad, CA, USA). Frozen tissue was ground in a 2 mL tube at 60 Hz for 60 s. One milliliter of TRIzol was added to the powder. The tissue was then ground at 55 Hz for 30 s and incubated at room temperature for 5 min. The homogenate was centrifuged at 12,000 rpm for 5 min. The supernatant was mixed vigorously with 0.2 mL chloroform for 30 s, incubated for 2–3 min, and centrifuged at 12,000 rpm for 15 min at 4 °C.

Approximately 400 μL of the aqueous phase was transferred to a new tube and mixed with 500 μL isopropanol. After 10 min at room temperature, RNA was pelleted at 12,000 rpm for 10 min at 4 °C. The pellet was washed with 1 mL of 75% ethanol and centrifuged at 12,000 rpm for 5 min at 4 °C. Residual ethanol was removed, and the pellet was dissolved directly in RNase-free water without complete drying.

RNA concentration and purity were measured with a NanoDrop One spectrophotometer (Thermo Scientific, Waltham, MA, USA). Integrity was assessed with an Agilent Fragment Analyzer 5400 (Agilent Technologies, Santa Clara, CA, USA). Each RNA preparation that passed quality assessment was divided into two aliquots. One aliquot was used for RNA-seq library construction, and the other was retained for qRT-PCR analysis.

### 2.3. Library Preparation and Sequencing

Poly(A)-selected mRNA libraries were prepared. Polyadenylated RNA was enriched with the NEBNext Poly(A) mRNA Magnetic Isolation Module (New England Biolabs, E7490L, Ipswich, MA, USA). The enriched RNA was fragmented with divalent cations using the NEBNext Ultra II RNA Library Prep Kit for Illumina (New England Biolabs, E7775). Double-stranded cDNA was synthesized with random oligonucleotide primers, purified, end-repaired, A-tailed, and ligated to sequencing adapters.

Fragments of approximately 400–500 bp were selected with AMPure XP beads (Beckman Coulter, Beverly, MA, USA). After PCR amplification and a second bead purification, library fragment size was checked by electrophoresis. Total library concentration was measured with PicoGreen (Invitrogen, Carlsbad, CA, USA), and effective library concentration was determined by qPCR. Libraries were pooled in equimolar amounts and subjected to paired-end sequencing on an Illumina NovaSeq X Plus instrument (Illumina, San Diego, CA, USA). The raw reads were deposited under BioProject PRJNA1506078; sample and run accessions are listed in Table S2.

### 2.4. Read Processing, Reference Alignment, and Expression Quantification

Raw FASTQ files were inspected with FastQC v0.11.9. Adapter-contaminated sequences and reads with a mean quality score below Q20 were removed with fastp v0.22.0. All downstream analyses used clean reads.

The chromosome-scale *Quercus mongolica* genome and its gene annotation (NCBI Assembly GCA_011696235.1) were used as the common reference [35]. An index was built with HISAT2 v2.1.0, and clean paired-end reads were aligned to the reference. The overall mapping rate, the number of uniquely mapped reads, and the proportion of reads assigned to annotated gene regions were used to assess reference compatibility across species.

Gene-level counts were obtained with HTSeq v0.9.1. Raw counts were used for differential-expression testing. Fragments per kilobase of transcript per million mapped reads (FPKM) were used to describe transcript abundance, assess the breadth of gene detection, and display candidate-gene expression profiles. The primary bioinformatic workflow was run on the Personalbio GenesCloud web platform (https://www.genescloud.cn/; Shanghai Personal Biotechnology Co., Ltd., Shanghai, China), and exported count and annotation files were used for the additional analyses described below.

### 2.5. Global Expression Structure and Differential Expression Analysis

Pairwise Pearson correlations between samples were calculated using FPKM values across genes and visualized with pheatmap v1.0.12. For the primary principal component analysis (PCA), genes with at least one mapped read across the 12 libraries were retained. Raw counts were divided by median-ratio size factors [36], transformed as log2(normalized count + 1), centered across samples within each gene, and subjected to PCA. The first two components were displayed in a two-dimensional score plot. Replicates were assessed using their positions in the PCA, pairwise correlations, read quality, and mapping statistics; none were excluded. For candidate-gene heatmaps, log2(FPKM + 1) values were standardized within each gene. PCA coordinates and normalization factors are provided in Table S3.

PCA was repeated to assess whether separation among species depended mainly on genes that were not consistently detected across species. The analysis was restricted to genes with FPKM ≥ 1 in at least two of the three replicates of every species. Raw counts for this shared-detection set were divided by median-ratio size factors [36], transformed as log2(normalized count + 1), centered across samples for each gene, and subjected to PCA. This analysis tested whether the species structure persisted within a set of genes detected in all four species. Shared-detection PCA coordinates and normalization factors are provided in Table S4.

Six pairwise contrasts were analyzed: Qf versus Qa, Qg versus Qa, Cs versus Qa, Qg versus Qf, Cs versus Qf, and Cs versus Qg. Raw counts were modeled with DESeq2 v1.38.3 [36]. Genes with |log2 fold change| ≥ 1 and a Benjamini–Hochberg-adjusted *p*-value < 0.05 were considered differentially expressed [37]. For clarity, results are described in terms of higher expression in the first or second species rather than by fold-change sign alone. Complete differential-expression results are provided in Table S5.

### 2.6. GO and KEGG Enrichment Analyses and GSEA

Gene Ontology (GO) enrichment analysis was performed with clusterProfiler v4.6.0 [38] using a hypergeometric test. Benjamini–Hochberg correction was applied across terms, and a false discovery rate (FDR) < 0.05 was considered significant. Complete GO results are provided in Table S6.

Kyoto Encyclopedia of Genes and Genomes (KEGG) over-representation analysis was performed with clusterProfiler v4.6.0 [38]. The background comprised genes that entered the analysis and had a valid KEGG annotation. To distinguish the direction of enrichment, genes with higher expression in the first or second species were tested separately with a hypergeometric test. Benjamini–Hochberg correction was applied across pathways, and FDR < 0.05 was considered significant. Complete KEGG over-representation results are provided in Table S7.

GSEA was also performed with clusterProfiler v4.6.0 [39]. For each contrast, all analyzed genes were ranked by the DESeq2 log_2_ fold change calculated as the second species relative to the first species. The normalized enrichment score (NES) was oriented so that a positive value indicated enrichment toward the second species and a negative value indicated enrichment toward the first species. Only gene sets with FDR < 0.05 were treated as primary evidence. Complete GSEA results are provided in Table S8.

For biological interpretation, a set of 14 KEGG pathways was grouped by relevance to carbon supply and allocation; growth investment and wall remodeling; or stress, redox, and metabolic homeostasis. These pathways were selected for the sensitivity summary; statistical testing was performed across all eligible KEGG pathways. The expression patterns described for each species summarize enrichment results and candidate annotations. Table S9 lists the pathway assignments, sensitivity-test eligibility, and representation in the candidate-to-pathway matrix.

To test whether the main pathway directions depended on genes detected inconsistently among species, we performed a directional resampling sensitivity analysis, restricting each ranked list to the same 12,223 genes used for the shared-detection PCA. A weighted running-sum statistic was calculated from DESeq2 log2 fold changes, with absolute fold change used as the hit weight. All KEGG sets containing 15–500 shared-detection genes were tested. Scores were standardized against the mean of the same-sign null distribution obtained from 1000 random gene sets matched for pathway size. Nominal *p*-values from that null distribution were adjusted across all eligible KEGG sets within each contrast by the Benjamini–Hochberg method. Because this resampling procedure differed from the primary GSEA implementation, it was used to assess direction and sensitivity-test significance rather than to reproduce the primary GSEA FDR values. Of the 14 pathways selected for the sensitivity display, 12 met the gene-set size criterion. Table S10 reports these pathways across all six contrasts. Retention of enrichment direction was summarized for associations with FDR < 0.05 in the primary GSEA; the complete sensitivity results are provided in Table S11.

### 2.7. Representative Candidate Gene Selection

Candidate genes were selected after the pathway-level analysis to form a representative panel. We considered differential expression in multiple contrasts and agreement between the direction of gene expression and pathway enrichment. Leading-edge membership, consistency among replicates, annotation reliability, and relevance to stem biology were also considered. Eleven candidates belonged to significant GSEA leading edges. *XTH23* was selected separately because it was differentially expressed in five contrasts and annotated for xyloglucan remodeling; it was not part of a significant leading edge. The representative panel contained three genes associated with each species: *PSBO*, *BMY1*, and *CHS1* for *Q. glauca*; *INVA*, *XTH23*, and *ABCG25* for *Q. acutissima*; *SAT3*, *UGE1*, and *PP2C37-like* for *Q. fabri*; and *ABCB11-like*, *RFS2*, and *CWINV1-like* for *C. sclerophylla*. Tables S12 and S13 document the statistical evidence and annotations supporting these selections. The candidate-to-pathway matrix includes every pathway with a significant leading-edge membership among the selected genes. This membership-based set is distinct from the pathway set used for the sensitivity display. The candidate-to-pathway matrix is provided in Table S14.

### 2.8. qRT-PCR Analysis

qRT-PCR was used to compare candidate-gene expression patterns with those obtained by RNA-seq, using aliquots from the same RNA extractions. The assays therefore used the same three biological replicates per species. Three technical reactions were performed for each biological replicate. *DCP5* was selected as a reference-gene candidate from the RNA-seq data because its abundance showed limited variation across the 12 libraries (60.42–73.77 FPKM). For each candidate, the same target-gene primer pair was used in all four species; species-specific primer pairs were used for the reference gene *DCP5* (gene-Qm026680). The predicted target amplicon lengths, based on the *Q. mongolica* reference CDS, were 132 bp for *PSBO*, 92 bp for *BMY1*, 110 bp for *CHS1*, 119 bp for *INVA*, 112 bp for *XTH23*, 125 bp for *ABCG25*, 91 bp for *SAT3*, 114 bp for *UGE1*, 149 bp for *PP2C37-like*, 99 bp for *ABCB11-like*, 161 bp for *RFS2*, and 105 bp for *CWINV1-like*. Table S15 provides the primer sequences, predicted product lengths, target-assay efficiencies, and *DCP5* Cq summaries. Predicted *DCP5* product lengths were 105 bp for *Q. glauca*, 105 bp for *Q. acutissima*, 97 bp for *Q. fabri*, and 101 bp for *C. sclerophylla*. *DCP5* product lengths were predicted from the reference CDS, incorporating SNP substitutions at primer-binding sites supported by the sample sequencing data. These are sequence-based product lengths; the predicted products and coordinates are provided in Table S15.

First-strand cDNA was synthesized from 1 μg of total RNA with HiScript III RT SuperMix for qPCR with genomic-DNA removal (Vazyme, Nanjing, China). qRT-PCR was performed on a QuantStudio 3 Real-Time PCR System (Thermo Fisher Scientific, Waltham, MA, USA) using ChamQ Universal SYBR qPCR Master Mix (Vazyme). Each 20 μL reaction contained 10 μL of 2× master mix, 0.4 μL each of forward and reverse primers (10 μM), 1 μL of diluted cDNA, and 8.2 μL of nuclease-free water. The cycling program was 95 °C for 30 s, followed by 40 cycles of 95 °C for 10 s and 60 °C for 30 s.

Cq values from the three technical reactions were averaged for each biological replicate. Target expression was normalized to the corresponding species-specific *DCP5* assay, with *Q. acutissima* as the calibrator. Relative expression was calculated using the 2^−ΔΔCq^ method [40]. Recorded target-assay efficiencies ranged from 94.04% to 102.42%. Across 108 reference-gene reactions per species, mean *DCP5* Cq values ranged from 22.31 to 22.66; these reactions used material from the same three seedlings and did not increase the biological sample size. *DCP5* RNA-seq abundance ranged from 60.42 to 73.77 FPKM across the 12 libraries. Reference-assay efficiencies were not independently recorded, so the comparison focused on observed expression ranks rather than calibrated differences in transcript abundance between species. Raw Cq values and biological-replicate calculations are provided in Tables S16 and S17.

### 2.9. Statistical Analysis

Statistical significance in the RNA-seq analyses was defined as a Benjamini–Hochberg-adjusted *p*-value or FDR < 0.05, as specified for each analysis. qRT-PCR profiles were summarized from three biological replicates. Consistency between RNA-seq and qRT-PCR was evaluated by comparing the species with the highest expression and the rank order of all four species for each gene.

## 3. Results

### 3.1. Sequencing Quality and Global Expression Structure

The 12 libraries generated 575,551,348 raw reads and 86,908,253,548 bases. Q30 values ranged from 96.55% to 96.82%, GC content from 43.29% to 43.91%, and clean-read retention from 98.66% to 98.85% (Table 1). Mean mapping rates against the *Q. mongolica* genome were 88.44% for *Q. fabri*, 59.09% for *Q. glauca*, 57.07% for *Q. acutissima*, and 46.82% for *C. sclerophylla*.

**Figure 1:**
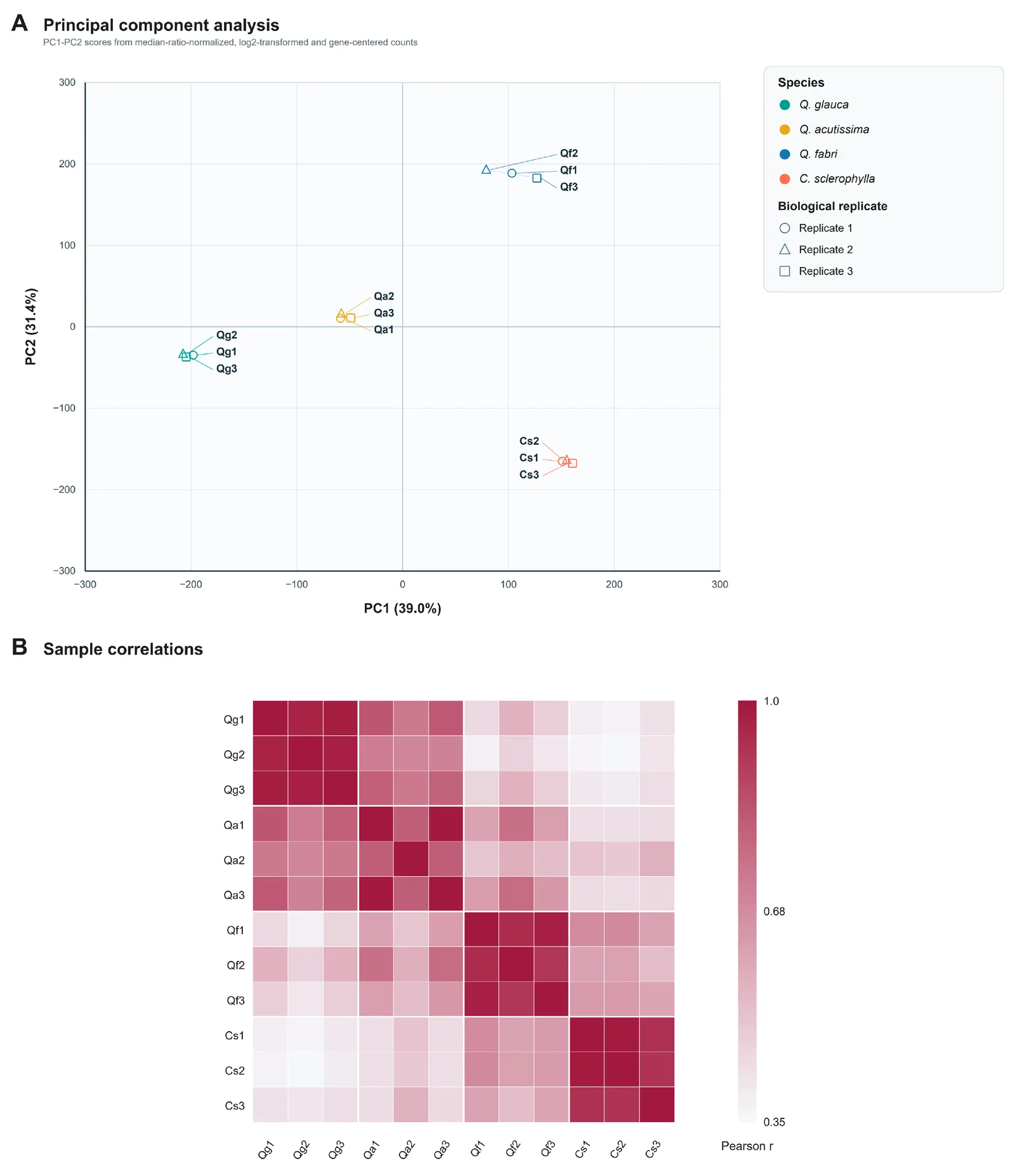
Transcriptome structure of the sampled juvenile stems. (**A**) Principal component analysis (PCA) scores for the first two principal components (PC1 and PC2), calculated from median-ratio-normalized counts after log2 transformation and gene centering. Colors identify species, and circles, triangles, and squares identify biological replicates 1, 2, and 3. (**B**) Pearson correlations among libraries. Species abbreviations: Qg, *Quercus glauca*; Qa, *Quercus acutissima*; Qf, *Quercus fabri*; Cs, *Castanopsis sclerophylla*.

The first two principal components explained 39.0% and 31.4% of the variance in the median-ratio-normalized count matrix, respectively, accounting for 70.4% in total. The samples formed four non-overlapping species clusters in PC1–PC2 space (Figure 1A; Table S3). Mean within-species Pearson correlation was 0.938, compared with 0.556 between species (Figure 1B). A sensitivity PCA based on 12,223 genes detected in every species also recovered four separate clusters; PC1 and PC2 explained 42.9% and 30.1% of the variance (Figure S1; Table S4). Thus, both the full dataset and the set of genes detected in all four species showed separation among species.

### 3.2. Differential Expression Identifies Pathways Related to Carbon Metabolism, Transport, and Regulation

**Figure 2:**
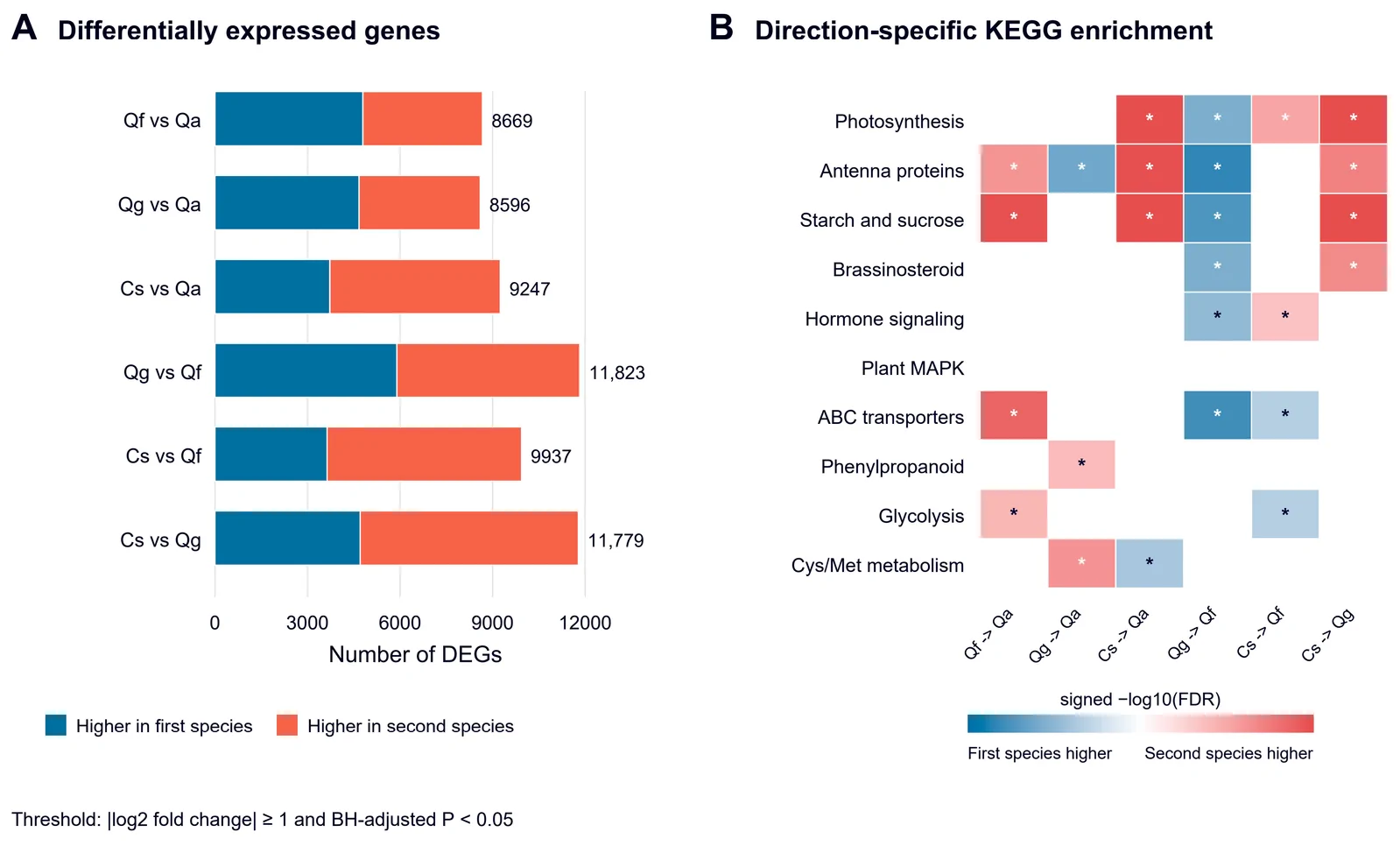
Differential-expression counts and direction-specific KEGG enrichment. (**A**) Number of genes with higher expression in the first or second species at |log2 fold change| ≥ 1 and BH-adjusted *p* < 0.05. (**B**) Directional enrichment strength for representative pathways. Blue indicates enrichment among genes with higher expression in the first species, and red indicates enrichment among genes with higher expression in the second species. Color intensity shows signed −log10(FDR); asterisks mark FDR < 0.05. Abbreviations: DEG, differentially expressed gene; KEGG, Kyoto Encyclopedia of Genes and Genomes; FDR, false discovery rate; ABC, ATP-binding cassette; MAPK, mitogen-activated protein kinase. Species abbreviations: Qg, *Q. glauca*; Qa, *Q. acutissima*; Qf, *Q. fabri*; Cs, *C. sclerophylla*.

**Figure 3:**
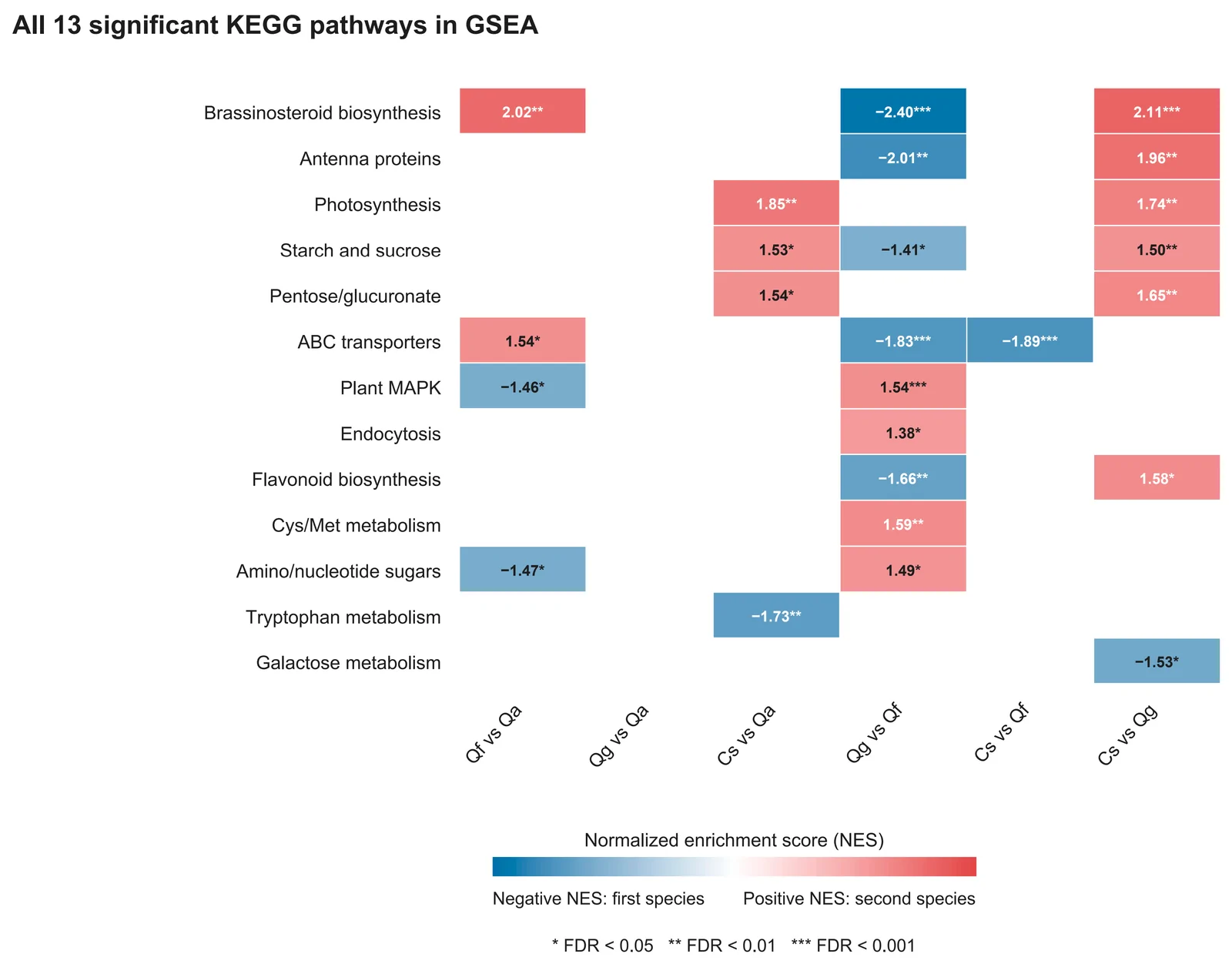
Gene set enrichment analysis (GSEA) results for the 13 KEGG pathways with at least one association at FDR < 0.05. Positive normalized enrichment score (NES) values indicate enrichment toward the second species in a contrast; negative values indicate enrichment toward the first species. Only cells with FDR < 0.05 are shown. Asterisks indicate significance: * FDR < 0.05; ** FDR < 0.01; *** FDR < 0.001. Species abbreviations: Qg, *Q. glauca*; Qa, *Q. acutissima*; Qf, *Q. fabri*; Cs, *C. sclerophylla*.

Reference-based analyses identified differentially expressed genes in all six pairwise contrasts (Figure 2A; Table 2). Totals ranged from 8596 genes in Qg–Qa to 11,823 in Qg–Qf. Direction-specific KEGG analysis identified 69 significant pathway-by-contrast enrichments, and GSEA identified 25 associations at FDR < 0.05 across 13 pathways (Figure 2B and Figure 3; Tables S7 and S8). These pathways included photosynthesis-related functions, carbohydrate and amino acid metabolism, nucleotide sugar metabolism, flavonoid biosynthesis, ATP-binding cassette (ABC) transport, endocytosis, and signaling.

Leading-edge extraction identified 980 unique genes across the 25 significant GSEA associations. Twenty-three genes contributed to more than one pathway (Tables S18 and S19). The sensitivity summary included 72 pathway-by-contrast associations across 12 pathways. Of these, 14 associations involving seven pathways had FDR < 0.05 in the primary GSEA. Thirteen of these 14 associations retained their direction, including all eight associations assigned to carbon supply and allocation. Six met BH-FDR < 0.05 in the sensitivity test (Figure S2; Tables S9–S11). The species-associated pathway results and candidate annotations are described below.

### 3.3. Photosynthesis, Starch Turnover, and Flavonoid Pathways in Q. glauca

In *Q. glauca*, the main expression pattern involved photosynthesis, carbohydrate metabolism, and specialized metabolism. In Cs–Qg, photosynthesis (NES = 1.745, FDR = 0.0061), photosynthesis-antenna proteins (NES = 1.959, FDR = 0.0017), starch and sucrose metabolism (NES = 1.497, FDR = 0.0031), and flavonoid biosynthesis (NES = 1.585, FDR = 0.0326) were enriched toward *Q. glauca*. Enrichment in the same direction was observed in Qg–Qf for antenna proteins (NES = −2.010, FDR = 0.0028), starch and sucrose metabolism (NES = −1.406, FDR = 0.0156), and flavonoid biosynthesis (NES = −1.663, FDR = 0.0084).

Three candidate genes were selected to represent this pattern. *PSBO* had a mean FPKM of 559.86 in *Q. glauca*, compared with 64.59 in *Q. acutissima*, 52.66 in *Q. fabri*, and 16.00 in *C. sclerophylla*. *BMY1* expression was also highest in *Q. glauca*, and the gene appeared in the leading edges of the starch and sucrose metabolism pathway in three contrasts. *CHS1* had a mean FPKM of 567.01 in *Q. glauca* and ≤19.87 in the other species. These candidates were annotated for photosystem function, starch degradation, and flavonoid biosynthesis, respectively.

### 3.4. Sugar Metabolism and ABC Transport in Q. acutissima

Sugar-metabolism and ABC-transporter pathways were enriched toward *Q. acutissima* in specific contrasts. In Cs–Qa, pentose and glucuronate interconversions (NES = 1.541, FDR = 0.0358) and starch and sucrose metabolism (NES = 1.526, FDR = 0.0118) favored *Q. acutissima*. Direction-specific enrichment also identified starch and sucrose metabolism in Cs–Qa (FDR = 1.22 × 10^−5^) and Qf–Qa (FDR = 1.09 × 10^−4^). ABC transporters were enriched toward *Q. acutissima* in Qf–Qa by both GSEA (FDR = 0.0158) and over-representation analysis (FDR = 3.12 × 10^−4^).

*INVA* had a mean FPKM of 1471.58 in *Q. acutissima* and belonged to the significant starch/sucrose and galactose leading edges. *XTH23* reached 984.30 FPKM in *Q. acutissima* and was differentially expressed in five contrasts. It was included as an additional wall-remodeling candidate on the basis of its annotation, despite lacking leading-edge support. *ABCG25* expression was highest in *Q. acutissima*, and the gene was present in an ABC-transporter leading edge. The enriched pathways and candidate annotations were associated with sugar metabolism, wall remodeling, and membrane transport at different levels of evidence.

### 3.5. Sulfur Amino Acid Metabolism, Nucleotide Sugar Metabolism, and Signaling in Q. fabri

Three significant GSEA pathways characterized the *Q. fabri*-associated expression pattern. In Qg–Qf, cysteine and methionine metabolism (NES = 1.590, FDR = 0.0072), amino sugar and nucleotide sugar metabolism (NES = 1.487, FDR = 0.0138), and plant mitogen-activated protein kinase (MAPK) signaling (NES = 1.540, FDR = 5.45 × 10^−4^) were enriched toward *Q. fabri*. Plant MAPK signaling also favored *Q. fabri* in Qf–Qa (NES = −1.465, FDR = 0.0158).

*SAT3* was most highly expressed in *Q. fabri* (mean FPKM, 344.59) and belonged to the cysteine-and-methionine leading edge. *UGE1* was most highly expressed in *Q. fabri* (mean FPKM, 202.75) and contributed to amino/nucleotide-sugar and galactose leading edges. *PP2C37-like* had a mean FPKM of 538.69 in *Q. fabri* and appeared in two plant-MAPK leading edges. These genes were annotated for sulfur amino acid metabolism, nucleotide sugar interconversion, and protein dephosphorylation, respectively.

### 3.6. ABC Transport and Galactose Metabolism in C. sclerophylla

ABC transport and galactose-related expression distinguished *C. sclerophylla*. ABC transporters were enriched toward *C. sclerophylla* in Cs–Qf (NES = −1.889, FDR = 1.19 × 10^−5^), whereas galactose metabolism favored *C. sclerophylla* in Cs–Qg (NES = −1.532, FDR = 0.0379). Candidate genes associated with these pathways were annotated for membrane transport, raffinose synthesis, and extracellular sucrose cleavage.

*ABCB11-like* had a mean FPKM of 32.99 in *C. sclerophylla*, compared with ≤2.28 in the three oaks, and contributed to two ABC-transporter leading edges. *RFS2* had a mean FPKM of 173.67 in *C. sclerophylla* and belonged to the galactose-metabolism leading edge. *CWINV1-like* expression was highest in *C. sclerophylla* (mean FPKM, 41.07). This gene was differentially expressed in all six contrasts and contributed to galactose and starch/sucrose leading edges. These results identify candidate transcripts associated with the *C. sclerophylla* pathway pattern.

### 3.7. Candidate Gene Evidence and qRT-PCR Analysis

**Figure 4:**
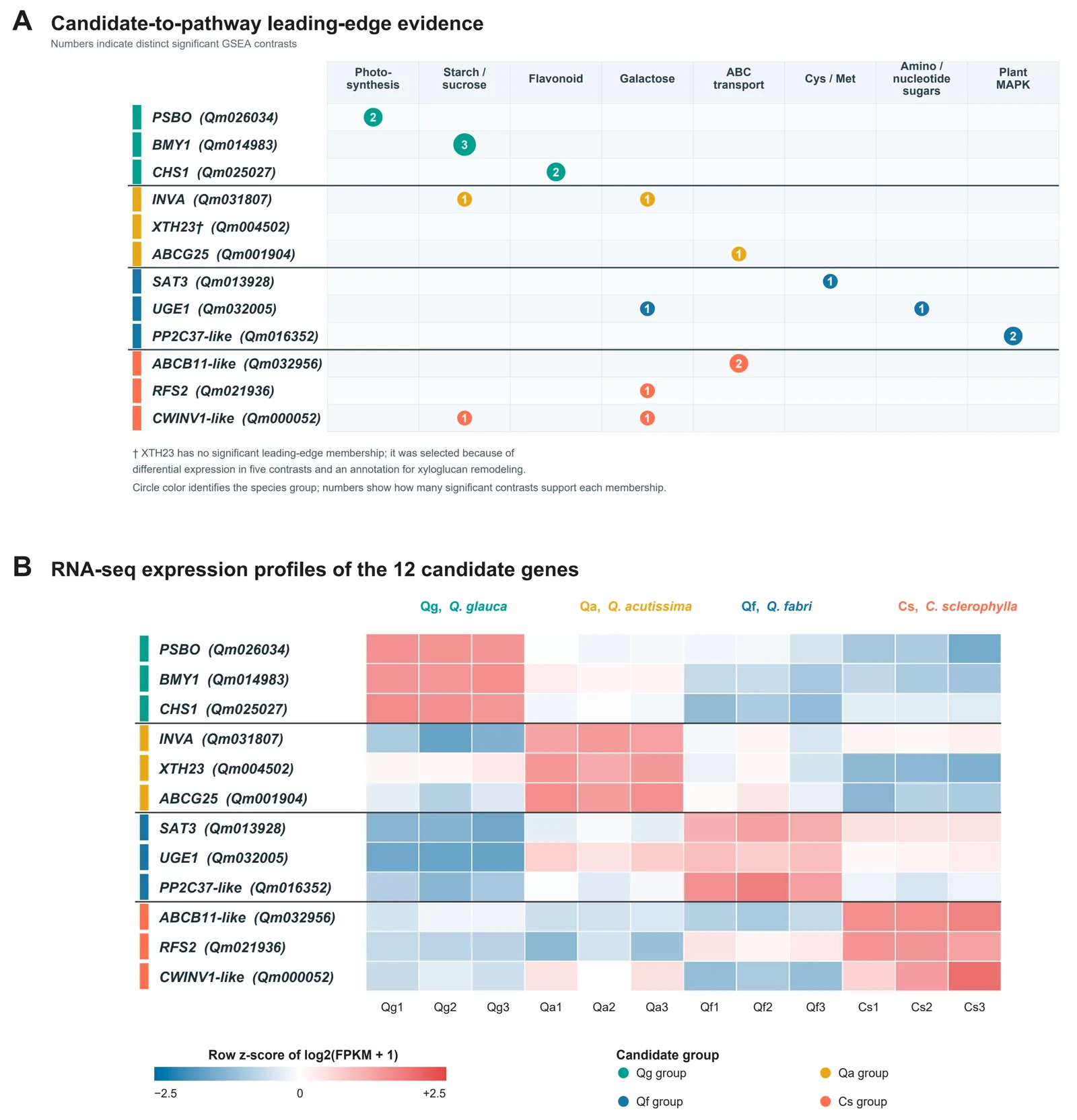
Pathway evidence and RNA-seq expression profiles of the 12 candidate genes. (**A**) Significant GSEA leading-edge memberships of the selected candidates across eight pathways. Numbers within circles indicate how many significant contrasts support each membership; larger circles represent more supporting contrasts, and circle colors identify the species-associated group. *XTH23* was selected using differential expression and annotation and has no leading-edge memberships. (**B**) Row-standardized log2(FPKM + 1) values across the 12 biological replicates. FPKM denotes fragments per kilobase of transcript per million mapped reads. Genes are arranged in four groups of three; the colored side strip identifies the associated species. Species abbreviations: Qg, *Q. glauca*; Qa, *Q. acutissima*; Qf, *Q. fabri*; Cs, *C. sclerophylla*.

The representative panel contained three genes associated with each species (Table 3; Figure 4). Eleven of the 12 genes belonged to at least one significant GSEA leading edge. The candidate-to-pathway matrix summarizes their membership in significant leading edges across eight pathways and records the number of contrasts supporting each membership. The matrix includes all pathways represented by significant leading-edge memberships among the selected genes (Figure 4A; Table S14). *XTH23* was included on the basis of differential expression and annotation and has no leading-edge memberships in the matrix. Figure 4B shows the RNA-seq profiles of all 12 candidates across the biological replicates.

**Figure 5:**
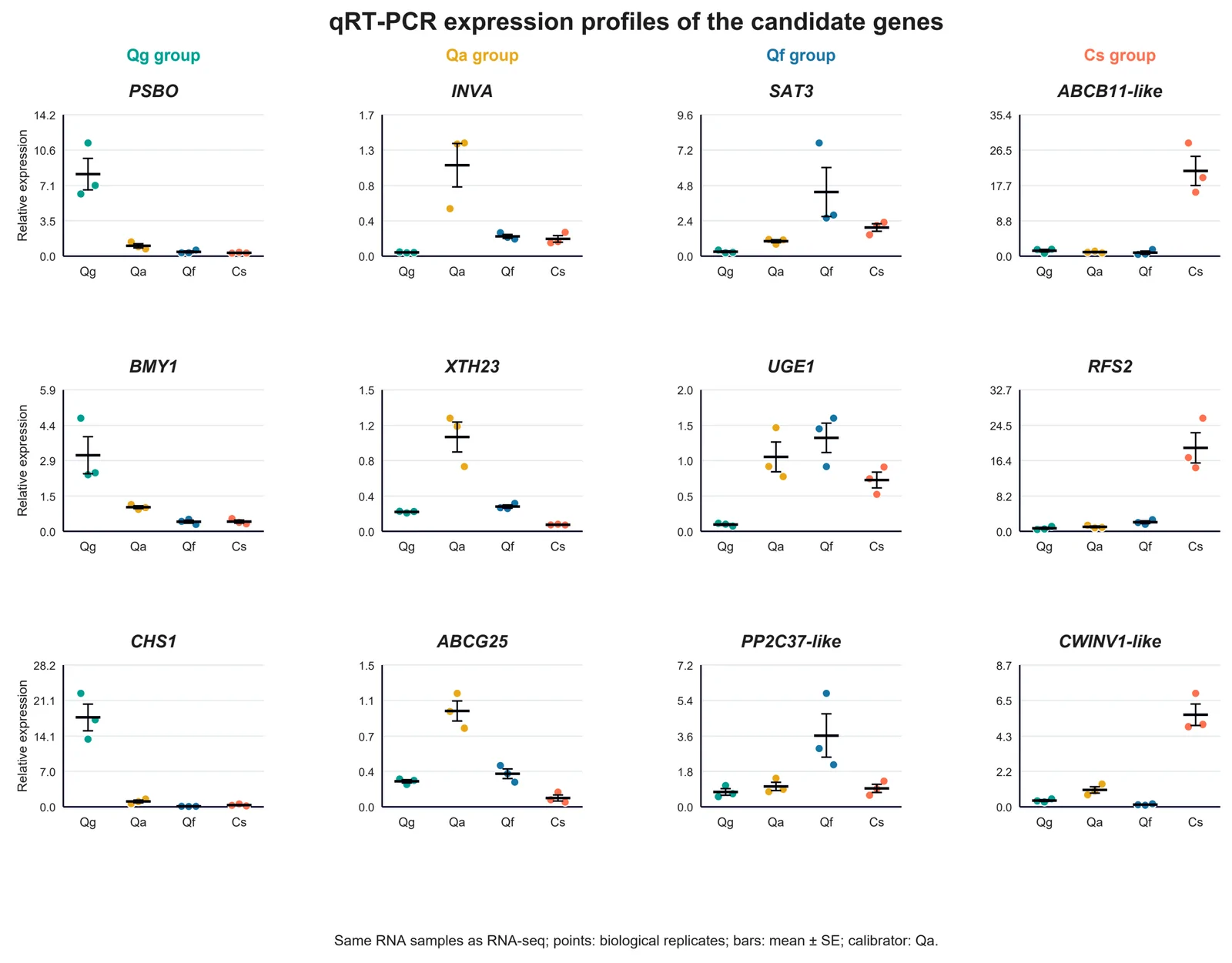
Quantitative reverse-transcription polymerase chain reaction (qRT-PCR) expression profiles of the candidate genes. Points represent three biological replicates per species from the same RNA extractions used for RNA-seq. Horizontal bars and error bars show the mean ± standard error after averaging the technical Cq values within each biological replicate. Relative expression was calculated by the 2^−ΔΔCq^ method using *Q. acutissima* as the calibrator. The four columns group candidates by their associated species (Qg, Qa, Qf, and Cs). Reference-assay efficiencies were not independently recorded; the values are used for expression-pattern comparison and are not calibrated cross-species fold changes. Species abbreviations: Qg, *Q. glauca*; Qa, *Q. acutissima*; Qf, *Q. fabri*; Cs, *C. sclerophylla*.

Mean *DCP5* Cq values ranged from 22.31 to 22.66 among species, and its RNA-seq abundance ranged from 60.42 to 73.77 FPKM across libraries (Table S15). RNA-seq and qRT-PCR identified the same species as having the highest expression for all 12 candidates (Figure 5; Tables S16 and S17). Eight genes had the same rank order across all four species in both assays: *PSBO*, *CHS1*, *ABCG25*, *SAT3*, *UGE1*, *PP2C37-like*, *ABCB11-like*, and *CWINV1-like*. For *BMY1*, *INVA*, *XTH23*, and *RFS2*, the rank order differed among the species with lower expression. These comparisons describe agreement between methods applied to the same biological samples.

**Figure 6:**
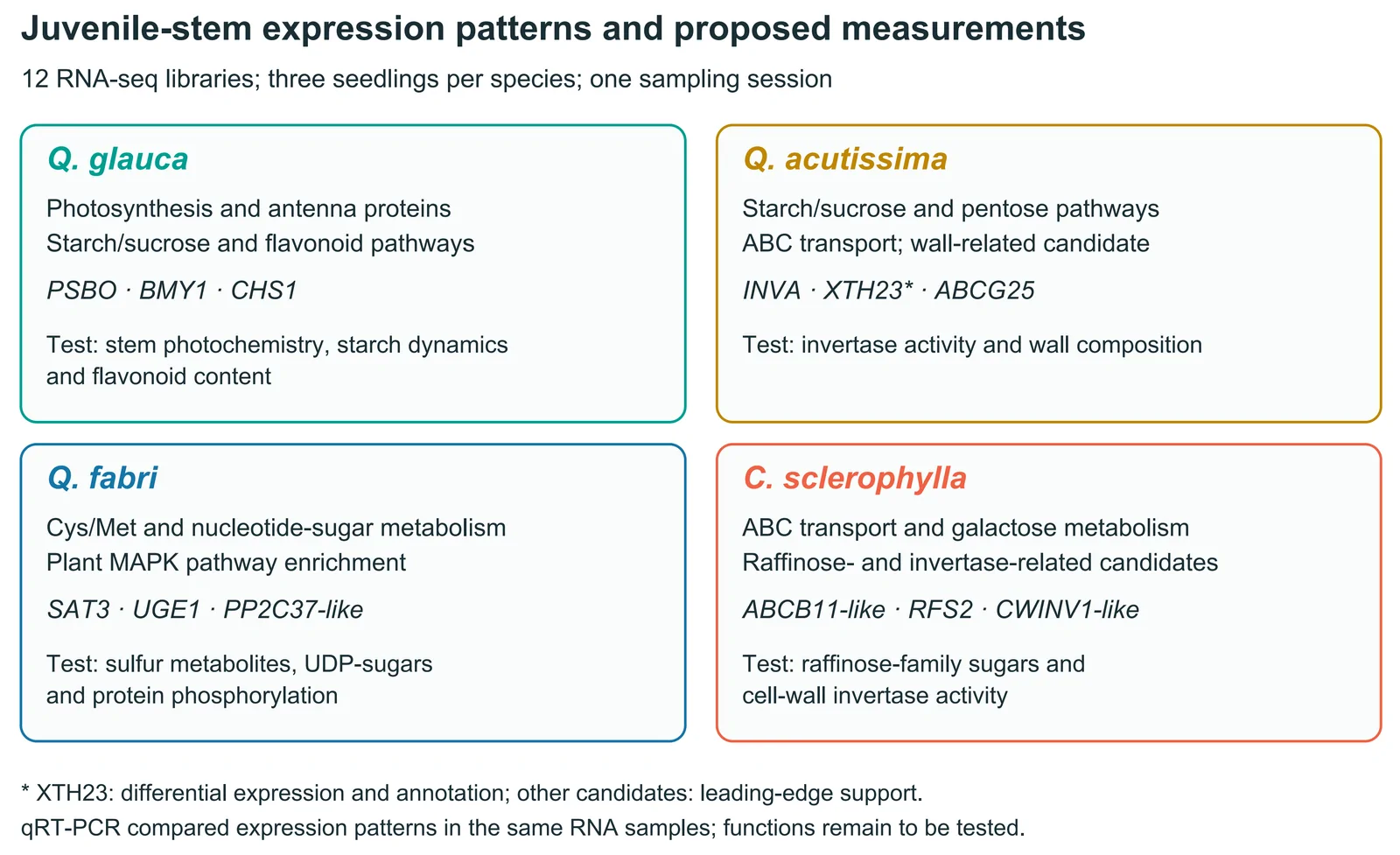
Juvenile-stem expression patterns and proposed measurements. The four groups summarize pathway enrichment and candidate annotations in the sampled species. Eleven candidates have significant GSEA leading-edge support; *XTH23* was selected using differential expression and annotation. Each group lists measurements for testing the functional interpretation. qRT-PCR compared expression patterns in the same biological samples. Species abbreviations: Qg, *Q. glauca*; Qa, *Q. acutissima*; Qf, *Q. fabri*; Cs, *C. sclerophylla*.

Figure 6 summarizes the pathway and candidate-gene evidence for each species.

## 4. Discussion

### 4.1. Distinct Combinations of Carbon Metabolism, Transport, and Regulation Among the Four Species

The sampled stems differed in the expression of genes associated with carbon metabolism, transport, and regulation. *Q. glauca* showed photosynthesis, starch turnover, and flavonoid pathway signals. *Q. acutissima* showed sugar metabolism and transport signals, together with the wall-remodeling candidate *XTH23*. *Q. fabri* showed enrichment of sulfur amino acid metabolism, nucleotide sugar metabolism, and MAPK signaling. *C. sclerophylla* showed ABC transport and galactose metabolism enrichment, with candidates annotated for raffinose synthesis and extracellular sucrose cleavage. These combinations provide a basis for examining how stem tissues use and distribute carbon. Transcript abundance reflects one level of regulation; metabolic flux, transport activity, and physiological function require direct measurements.

Transcriptional divergence among oak species has also been reported under soil-water constraints [12]. Here, we examined juvenile stems without an imposed stress treatment; the main differences among species involved carbon-related and transport pathways.

Each sample represented all tissues at the sampled stem position. Transcript and metabolite atlases show spatial differences among phloem, cambium, expanding xylem, and mature wood [1,2,3,5,24,25,26,27]. Sampling at 1 m does not establish that these tissues were present in equal proportions or at equivalent developmental stages in the four species. For example, a photosynthesis-related signal could reflect the proportion of chlorophyll-containing tissue, expression within that tissue, or both. Wall-related signals could similarly reflect differences in cambial activity or developing xylem. The species-associated expression patterns therefore describe integrated whole-stem profiles; the present sampling design cannot distinguish these contributions.

### 4.2. Carbon Acquisition and Use in Q. glauca and Q. acutissima

*Q. glauca* showed photosynthesis and antenna-protein enrichment together with high *PSBO* expression. PsbO stabilizes the oxygen-evolving complex of photosystem II, and its isoforms make different contributions to photosystem performance [41]. Woody-tissue photosynthesis can contribute to growth and bud development in young plants [4]. This expression pattern provides a basis for comparing stem photochemical activity among the four species, with chlorophyll content and tissue anatomy measured alongside fluorescence.

The *Q. glauca* candidate group also contained *BMY1* and *CHS1*. Beta-amylases participate in starch degradation [18,42], whereas chalcone synthase catalyzes an early step in flavonoid biosynthesis [43]. Their expression, together with pathway enrichment, suggests that starch turnover and flavonoid metabolism warrant examination alongside photosynthetic activity. Diel starch measurements and flavonoid profiling would assess whether these transcript patterns correspond to differences in metabolite pools.

In *Q. acutissima*, high *INVA* expression accompanied enrichment of starch/sucrose and pentose/glucuronate pathways. The annotation of *XTH23* links this candidate to xyloglucan remodeling, a process involved in wall loosening and expansion [20,21]. *ABCG25* contributed to an ABC transporter leading edge. The characterized *Arabidopsis ABCG25* protein exports ABA [44], but the substrate of the *Q. acutissima* candidate has not been established. Invertase assays and wall-composition measurements would test the metabolic interpretation, while transporter localization and substrate assays would address the transport component.

### 4.3. Sulfur, Nucleotide-Sugar, and Signaling-Related Expression in Q. fabri

The *Q. fabri* pathway pattern included sulfur amino acid metabolism. Serine acetyltransferase produces O-acetylserine, a precursor of cysteine. In *Arabidopsis*, reducing mitochondrial *SAT3* decreases O-acetylserine formation and sulfur incorporation into cysteine and glutathione [45]. The candidate in this study is annotated as a chloroplast-associated serine acetyltransferase. Its high expression and membership in a cysteine- and methionine-leading edge support its selection as a candidate for this pathway. Its localization and isoform-specific activity remain to be determined.

*UGE1* and *PP2C37-like* represent additional components of the *Q. fabri* pattern. Plant UDP-glucose/UDP-galactose epimerases interconvert nucleotide sugars used in metabolism and wall biosynthesis; individual isoforms differ in their kinetics and expression [46]. *PP2C37-like* is annotated as a phosphatase and occurs in two plant-MAPK leading edges. Some plant PP2Cs regulate ABA and stress responses [47], but pathway membership alone does not establish the substrates or activity of this candidate. MAPK signaling, redox regulation, and transcriptional control provide context for interpreting the broader expression pattern [29,30,31,32,33,34]. A transcriptome–proteome study of *Q. fabri* axillary-bud outgrowth also implicated sucrose metabolism [14]. Measuring sulfur metabolites, UDP-sugars, and protein phosphorylation would help assess the relationships suggested by the stem transcript profiles.

### 4.4. Transport- and Sugar-Related Expression in C. sclerophylla

*C. sclerophylla* showed enrichment of ABC transport and galactose metabolism. Raffinose synthases transfer galactosyl units to sucrose to produce raffinose, and raffinose-family oligosaccharides participate in carbon storage, transport, and stress responses [48]. *RFS2* expression was highest in *C. sclerophylla*, and the gene was present in a significant galactose-metabolism leading edge. Measuring raffinose-family sugars would help determine whether this expression pattern is associated with changes in sugar accumulation.

Cell-wall invertases cleave sucrose in the apoplast and can affect source–sink allocation [19,49]. *CWINV1-like* was differentially expressed in six contrasts and belonged to leading edges in two pathways. These results identify a candidate for testing extracellular sucrose cleavage in *C. sclerophylla*. *ABCB11-like* is a candidate for further study of transport. Several characterized plant ABCB proteins transport auxin [50], but the substrate of the *C. sclerophylla* protein is unknown. Measurements of raffinose-family sugars, cell-wall invertase activity, and transporter function would help assess whether the processes represented by these candidates are coordinated.

### 4.5. Candidate Gene Evidence and qRT-PCR Expression Support

Eleven candidates were supported by significant GSEA leading-edge membership, whereas *XTH23* was selected from differential-expression and annotation evidence. All 12 were differentially expressed in at least four contrasts. For every candidate, qRT-PCR and RNA-seq identified the same species as having the highest expression. Eight genes also showed the same rank order across all four species. Because both assays used aliquots from the same RNA extractions, the agreement reflects a comparison between methods within the sampled seedlings, rather than independent biological validation. Differences in primer binding and amplification efficiency among species can also affect expression ranks; similar *DCP5* Cq values alone do not establish assay equivalence. The candidate profiles should therefore be evaluated in additional biological samples with validated assays when testing their physiological roles.

Figure 6 and Table S20 distinguish the observed transcript patterns from the physiological hypotheses. The proposed measurements address stem photochemistry and starch dynamics in *Q. glauca*, invertase activity and wall composition in *Q. acutissima*, sulfur metabolites and UDP-sugars in *Q. fabri*, and raffinose-family sugars and cell-wall invertase activity in *C. sclerophylla*.

### 4.6. Limitations and Future Work

This study examined three seedlings per species at a single age and sampling time under shared growth conditions. This design identifies expression differences within the sampled material but cannot establish whether they persist across populations, seasons, or developmental stages. Whole-stem expression profiles also reflect both gene regulation and possible differences in tissue composition. Additional seedlings, repeated sampling, and anatomical or tissue-resolved measurements would determine how broadly the observed patterns apply.

Reads from all four species were mapped to the *Q. mongolica* reference, with mean mapping rates ranging from 46.82% in *C. sclerophylla* to 88.44% in *Q. fabri*. Sequence divergence from the reference can affect which genes are recovered, their estimated fold changes, and the number of detected DEGs [51,52,53]. The PCA based on 12,223 genes detected in all four species still separated the sampled species. Thirteen of the 14 associations in the sensitivity summary that were significant in the primary GSEA retained their enrichment direction, and six met the sensitivity-test BH-FDR threshold. These analyses assess dependence on gene detection; they do not establish one-to-one orthology or remove alignment bias within the retained genes. The DEG totals therefore reflect both biological differences and reference compatibility. Species-matched sequence resources and an analysis restricted to one-to-one orthologs would be needed to evaluate gene-level differences with less bias from differences in reference compatibility.

## 5. Conclusions

Under the sampled conditions, the four species showed distinct whole-stem expression patterns. *Q. glauca* showed photosynthesis, starch turnover, and flavonoid pathway signals. *Q. acutissima* showed sugar metabolism and transport signals, with an additional candidate for wall remodeling. *Q. fabri* showed sulfur amino acid metabolism, nucleotide sugar metabolism, and MAPK signaling enrichment. *C. sclerophylla* showed ABC transport and galactose metabolism enrichment, with candidates annotated for raffinose synthesis and extracellular sucrose cleavage.

The 12 candidates connect these pathway results and functional annotations to specific transcripts. Eleven were present in at least one significant GSEA leading edge. Applied to the same biological samples, RNA-seq and qRT-PCR identified the same species as having the highest expression for every candidate. Eight genes also showed the same rank order across all four species. These results guide further studies of stem photochemistry, carbohydrate turnover, wall composition, sulfur metabolism, and transport.

## Figures and Tables

**Table 1:** Mean RNA-seq quality and mapping metrics for each species (three biological replicates per species). Q30 is the percentage of bases with a Phred quality score of at least 30; GC denotes guanine plus cytosine content. Species abbreviations: Qg, *Quercus glauca*; Qa, *Quercus acutissima*; Qf, *Quercus fabri*; Cs, *Castanopsis sclerophylla*.

Species	Raw Reads per Library	Q30 (%)	GC (%)	Clean-Read Retention (%)	Mapping Rate (%)
*Q. glauca*	46,506,256	96.70	43.36	98.74	59.09
*Q. acutissima*	50,599,032	96.62	43.77	98.71	57.07
*Q. fabri*	46,171,885	96.73	43.62	98.75	88.44
*C. sclerophylla*	48,573,277	96.75	43.37	98.79	46.82

**Table 2:** Differentially expressed genes in the six pairwise contrasts. Species abbreviations: Qg, *Q. glauca*; Qa, *Q. acutissima*; Qf, *Q. fabri*; Cs, *C. sclerophylla*.

Contrast	Higher in First Species	Higher in Second Species	Total
*Q. fabri* vs. *Q. acutissima*	4795	3874	8669
*Q. glauca* vs. *Q. acutissima*	4671	3925	8596
*C. sclerophylla* vs. *Q. acutissima*	3724	5523	9247
*Q. glauca* vs. *Q. fabri*	5896	5927	11,823
*C. sclerophylla* vs. *Q. fabri*	3635	6302	9937
*C. sclerophylla* vs. *Q. glauca*	4708	7071	11,779

**Table 3:** Candidate genes representing the four species-associated expression patterns. Species abbreviations: Qg, *Q. glauca*; Qa, *Q. acutissima*; Qf, *Q. fabri*; Cs, *C. sclerophylla*.

Gene ID	Symbol	Species	Annotation	DEG Contrasts	Leading Edges
gene-Qm026034	*PSBO*	Qg	Photosystem II oxygen-evolving complex	5	2
gene-Qm014983	*BMY1*	Qg	Starch mobilization	5	3
gene-Qm025027	*CHS1*	Qg	Flavonoid biosynthesis	5	2
gene-Qm031807	*INVA*	Qa	Acid invertase and sucrose cleavage	5	2
gene-Qm004502	*XTH23*	Qa	Xyloglucan remodeling	5	0
gene-Qm001904	*ABCG25*	Qa	ABC transport	5	1
gene-Qm013928	*SAT3*	Qf	Serine acetyltransferase	5	1
gene-Qm032005	*UGE1*	Qf	UDP-sugar epimerization	4	2
gene-Qm016352	*PP2C37-like*	Qf	PP2C-family phosphatase	4	2
gene-Qm032956	*ABCB11-like*	Cs	ABC transport	5	2
gene-Qm021936	*RFS2*	Cs	Raffinose synthesis	5	1
gene-Qm000052	*CWINV1-like*	Cs	Cell-wall invertase	6	2

## Data Availability

The raw paired-end RNA-seq reads have been deposited in the NCBI Sequence Read Archive under BioProject PRJNA1506078 (https://www.ncbi.nlm.nih.gov/bioproject/PRJNA1506078, accessed on 1 August 2026). The 12 BioSample and SRA Run accessions are listed in Table S2. The supplementary tables provide processed differential-expression and enrichment results, evidence for candidate selection, shared-detection sensitivity results, and qRT-PCR measurements.

## Supplementary Materials

The following supporting information can be downloaded at: https://www.mdpi.com/article/10.3390/genes17091139/s1. Figure S1: Shared-detection sensitivity PCA; Figure S2: Directional resampling sensitivity analysis of the selected pathways; Table S1: Plant materials and sample metadata; Table S2: RNA-seq library quality, mapping, and accession information; Table S3: Main PC1–PC2 coordinates and normalization factors; Table S4: Shared-detection PCA coordinates and normalization factors; Table S5: Complete differential-expression results; Table S6: Complete GO enrichment results; Table S7: Complete KEGG over-representation results; Table S8: Complete GSEA results; Table S9: Pathway sets used in the sensitivity summary and candidate matrix; Table S10: Selected pathway evidence across the primary and sensitivity analyses; Table S11: Directional resampling sensitivity results; Table S12: Evidence for the 12 candidate genes; Table S13: Additional evidence for candidate-gene selection; Table S14: Candidate-to-pathway matrix used in Figure 4A; Table S15: qRT-PCR primer sequences, predicted amplicons, and DCP5 Cq summaries; Table S16: Raw qRT-PCR Cq measurements; Table S17: Processed qRT-PCR relative expression; Table S18: Significant GSEA leading-edge memberships; Table S19: Recurrent GSEA leading-edge genes; Table S20: Transcriptome-supported patterns and testable physiological hypotheses.
