# Supplementary material for: Comparative Transcriptomics Reveals Divergent Expression Patterns Related to Carbon Metabolism, Transport, and Regulation in Juvenile Stems of Four Fagaceae Species
Source: Genes (Basel). 2026 Sep 17;17(9):1139. doi: 10.3390/genes17091139 (PMC13606254; doi:10.3390/genes17091139)
Supplement: Supplementary file 1 [file genes-17-01139-s001.zip › Figure_S1. Shared-detection sensitivity PCA.pdf]

Sensitivity PCA using genes consistently detected in all four species

12,223 genes; FPKM ≥ 1 in at least 2 of 3 replicates per species

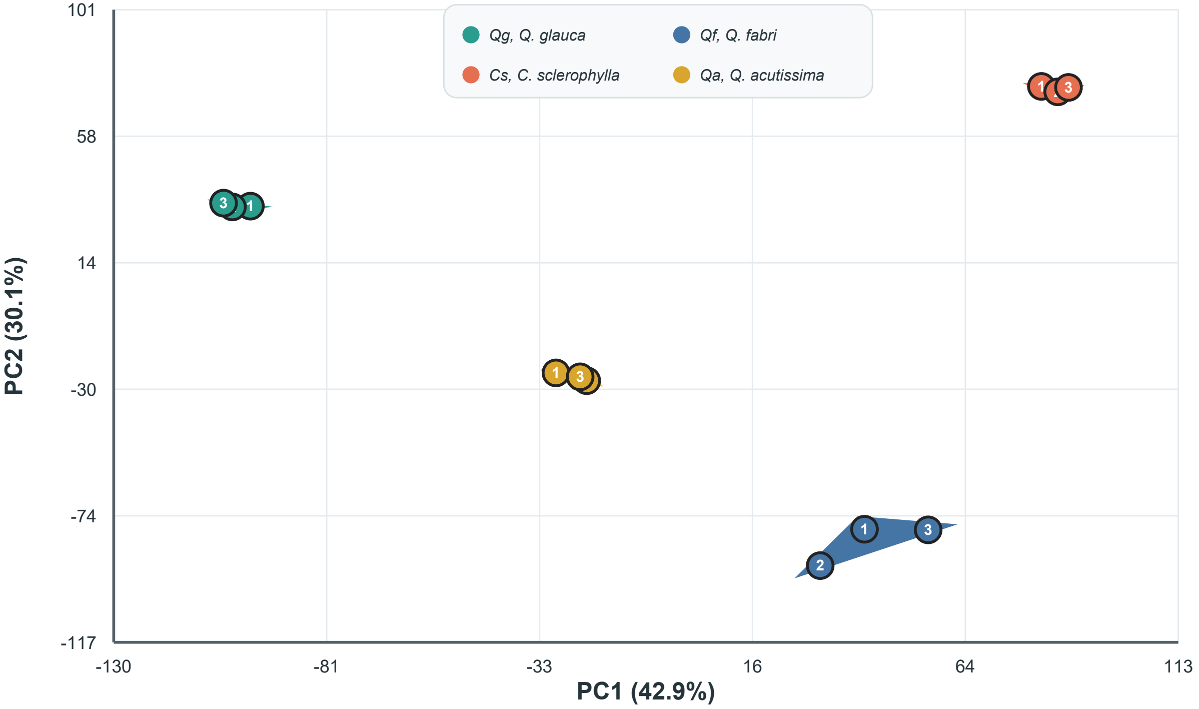

Replicate envelopes are descriptive.
The shared-detection analysis tests whether species-level structure persists after inconsistently detected genes are removed.
