# Supplementary material for: Comparative Transcriptomics Reveals Divergent Expression Patterns Related to Carbon Metabolism, Transport, and Regulation in Juvenile Stems of Four Fagaceae Species
Source: Genes (Basel). 2026 Sep 17;17(9):1139. doi: 10.3390/genes17091139 (PMC13606254; doi:10.3390/genes17091139)
Supplement: Supplementary file 1 [file genes-17-01139-s001.zip › Figure_S2. Directional resampling sensitivity analysis of the selected pathways.pdf]

Directional resampling sensitivity analysis

Rankings restricted to 12,223 genes consistently detected in all four species

Positive NES favors the second species in each contrast; negative NES favors the first

FUNCTIONAL CATEGORY	PATHWAY	Qf vs Qa	Qg vs Qa	Cs vs Qa	Qg vs Qf	Cs vs Qf	Cs vs Qg
Carbon supply and allocation	Photosynthesis	1.63	-1.84 *	2.30 *	-2.23 *	1.87	2.54 *
	Starch and sucrose metabolism	1.48	1.46	1.46	-1.23	1.17	1.32
	ABC transporters	1.18	1.08	-1.18	-1.23	-1.22	-1.11
Growth investment and wall remodeling	Plant hormone signal transduction	-0.93	1.02	1.30	1.01	1.29	1.37
	Pentose and glucuronate interconversions	0.91	-1.03	1.90 *	-1.12	1.60	1.73 *
	Protein processing in endoplasmic reticulum	0.97	1.85 *	1.01	1.47 *	0.88	-1.44
Stress, redox, and metabolic homeostasis	MAPK signaling pathway – plant	-0.94	1.64 *	1.10	1.48 *	1.12	-1.27
	Glutathione metabolism	-1.09	0.86	0.97	-0.79	1.09	1.10
	Cysteine and methionine metabolism	-1.75	1.54	-1.55	1.66 *	0.86	-1.72 *
	Glycerophospholipid metabolism	-1.05	1.38	-0.92	1.58 *	0.86	-1.35
	Glycolysis/gluconeogenesis	1.08	1.14	-1.05	0.99	-1.18	-1.49
	Tryptophan metabolism	1.01	-0.96	0.85	-1.28	-0.94	1.11

NES (First species ← → Second species)

* FDR < 0.05; ** FDR < 0.01; *** FDR < 0.001

Gene-set sizes were restricted to 15–500 genes. FDR was adjusted across all eligible KEGG sets within each contrast.
